# Monkeypox: From Emerging Trends to Therapeutic Concerns

**DOI:** 10.7759/cureus.58866

**Published:** 2024-04-23

**Authors:** Kiran G Piparva, Nilesh Fichadiya, Tejal Joshi, Shahenaz Malek

**Affiliations:** 1 Department of Pharmacology, All India Institute of Medical Sciences (AIIMS) Rajkot, Rajkot, IND; 2 Department of Preventive and Social Medicine, Pandit Deendayal Upadhyay (PDU) Government Medical College, Rajkot, IND; 3 Department of Microbiology, Pandit Deendayal Upadhyay (PDU) Government Medical College, Rajkot, IND; 4 Department of Pharmacology, Government Medical College, Surat, IND

**Keywords:** tecovirimat, vigiv, oxpv, pheic, hmxpv, mpox, bcv

## Abstract

Monkeypox is a zoonotic viral disease. Monkeypox was first reported in humans about 54 years ago. Prior to the global outbreak, monkeypox was endemic to the rainforests of central and western African countries. In the last three years, increasing numbers of human monkeypox have been reported from various countries. Responding to the severity, monkeypox was declared a Public Health Emergency of International Concern by the World Health Organization. In the absence of approved drugs or clinical studies, repurposed drugs and therapeutic medical countermeasures effective against other orthopoxviruses have been utilized to treat severe human monkeypox cases. Currently, clinical trials are underway exploring the potential therapeutic effectiveness of tecovirimate in human monkeypox cases. Monoclonal antibodies, IFN-β, resveratrol, and 15 triple-targeting FDA-approved drugs represent potential new drug targets for human monkeypox, necessitating further research.

## Introduction and background

Monkeypox (mpox) is a zoonotic disease caused by a double-stranded DNA virus that belongs to the orthopoxvirus (OPXV) genus of the poxviridae family [1]. Mpox was first isolated in 1958 from pox lesions during an outbreak of vesicular disease among captive cynomolgus macaques, monkeys imported from Singapore into Denmark for polio-vaccine-related research [2]. In 1959 the virus was first identified as a causative agent for pox infection outbreak among cynomolgus monkeys at the State Serum Institute, Copenhagen, Denmark [3]. Hence, the term "monkeypox" originated from the virus's first detection among monkeys in a research laboratory. Following this discovery, mpox was sporadically found in the tropical rainforests of central and western Africa [4]. Twelve years later, the first human monkeypox virus (hMPXV) infection was detected in 1970 in a nine-month-old child in the Democratic Republic of Congo (DRC) [5]. There were sporadic outbreaks in central and western African countries, typically originating from contact with wildlife reservoirs (particularly rodents) [6]. Prior to the global outbreak, from 1970 to 2003, monkeypox cases and outbreaks were mostly confined to the rainforests of central and western African countries where the virus is endemic [1,7].

There are two genetically distinct clades of human monkeypox virus, Clade I & Clade II (subtypes IIa & IIb) [1]. Clade I causes severe disease and higher mortality, especially in children, and is responsible for mpox cases in the DRC [8]. In DRC, the clade-dependent case fatality rates of 1 to 10% [9,10]. Various studies focused on identifying the natural reservoirs or hosts of the monkeypox virus and observed a range of animals including squirrels, rodents (Cricetomys and Graphiurus,) and the elephant shrew [11]. Several animal species, mostly rodents and non-human primates, have been confirmed to be susceptible to the virus after multiple investigations [12,13]. The virus may be transmitted from animals to humans by direct contact with diseased parts or body fluids of infected animals, scratching or biting by animals, eating meat from infected animals, and contact with contaminated objects. The current ongoing outbreak has disproportionately affected men who are gay or bisexual and other men who have sex with men, which suggests amplification of transmission through sexual networks [3,5,6] and many studies reported that also [13-15]. However, the role of semen or vaginal fluids in the transmission remains unclear and requires further research [16]. Virus is also transmitted from mother to child via the placenta, or close contact during and after birth [17]. The re-emergence of mpox might be due to a combination of environmental and ecological changes, animal or human movement, cessation of routine smallpox vaccination since eradication in 1980, improvements in disease detection and diagnosis, and genetic changes in the virus [14]. This review article focuses on reemerging trends about hMXPV and exploring the contemporary therapeutic strategies available for its treatment.

## Review

Monkeypox outbreaks globally

**Figure 1:**
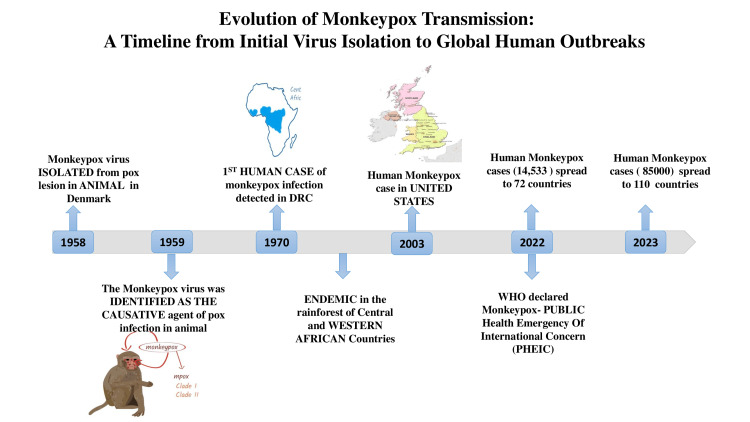
Monkeypox: journey from virus isolation to global outbreak. The figure illustrates the temporal progression of monkeypox, from its initial isolation in 1958 to a notable outbreak in 2022 [15-20]. DRC: Democratic Republic of Congo, PHEIC: Public Health Emergency of International Concern.

The first human case of mpox (hMPXV) outside Africa was documented in the USA in the year 2003 when a shipment of exotic animals was imported from Ghana. Since then, sporadic outbreaks have been noted across the globe, all having traceable origins in the endemic African regions [15]. The United Kingdom Health Security Agency (UKHSA) confirmed the first case of the hMPXV on May 7, 2022, in an individual returning from Nigeria. This individual later transmitted the infection to their family members [16]. Between January 1 to June 15, 2022, a total of 2103 confirmed cases and one death have been reported to WHO from 42 countries in five WHO regions [17]. In May 2022, a Vmulti-country outbreak of mpox was declared [18,19]. WHO declared mpox as a Public Health Emergency of International Concern (PHEIC) on July 23, 2022 [20]. From January 2022 to February 2023, more than 85,000 cases and 100 deaths were reported in 110 countries, the vast majority of high-income countries outside the African continent, propagated almost exclusively by human-to-human transmission [21,19] (Figure 1).

**Figure 2:**
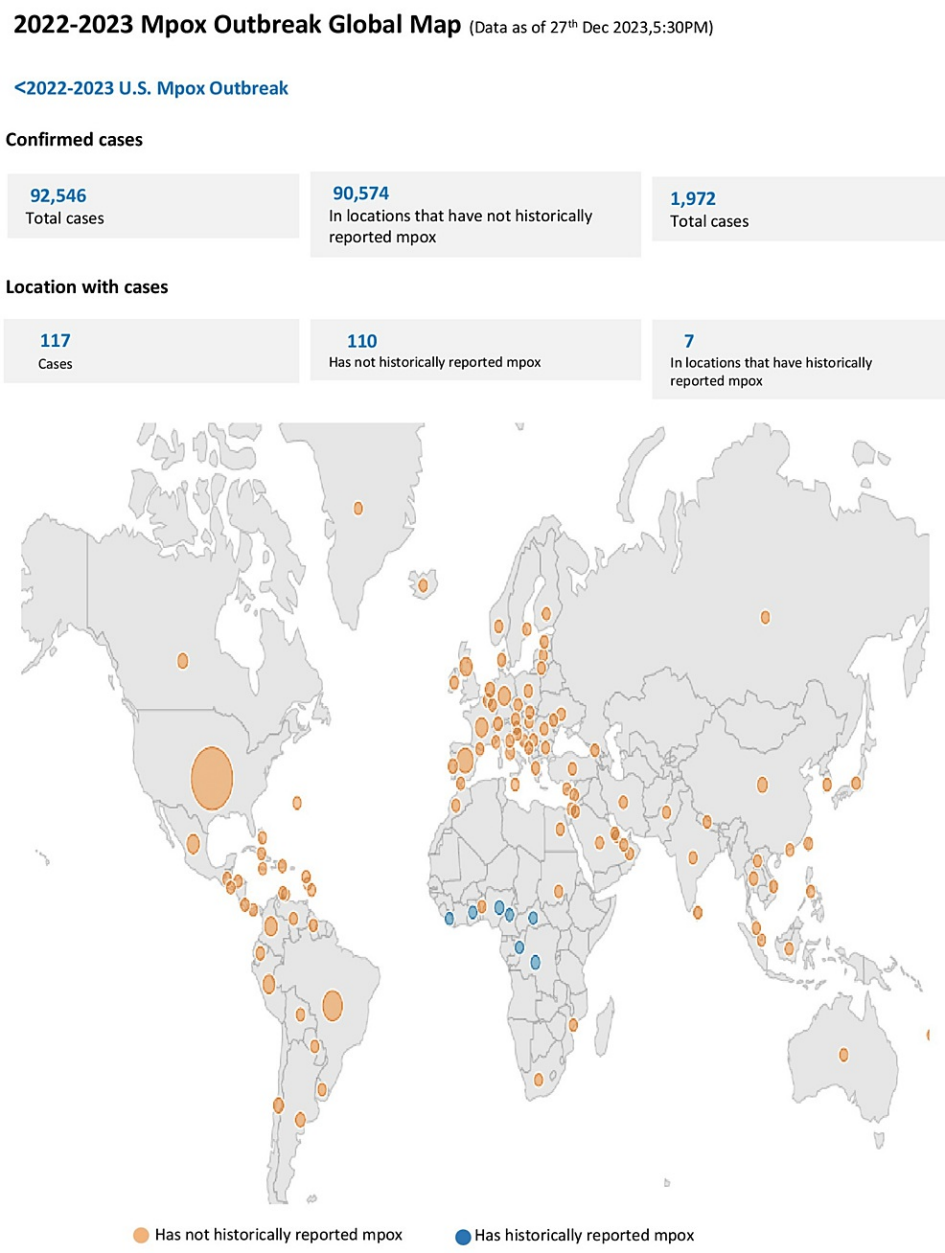
Global outbreak of monkeypox during 2022-2023. This figure details outbreaks of mpox, showing confirmed cases in the regions of America, Africa, Europe, Southeast Asia, and the Eastern Mediterranean during 2022-2023 [23]. Source: Centers for Disease Control and Prevention (CDC). (Permission has been obtained.)

According to geographic distribution, the regions of America, Africa, Europe, Southeast Asia, and the Eastern Mediterranean account for most of the confirmed cases of hMPXV [22-24] (Figure 2).

hMPXV cases in India

In India, the first case of hMPXV was reported from Kerala on July 14, 2022 (returned from the United Arab Emirates four days before the development of symptoms), and subsequent reporting of cases within intervals of one to two days in Delhi and Kerala primarily. Many of them had a history of international travel within the last 21 days, and while the majority affected were men, only two of them were women (aged 31 and 22 years). The first death due to mpox was reported from Thrissur, Kerala who was a 22-year-old man who returned from the United Arab Emirates. Notably, the deceased did not have symptoms of mpox but rather showed symptoms of encephalitis and fatigue [25-27]. As of September 18, 2022, the Indian Council of Medical Research (ICMR) had received 96 samples of suspected mpox cases from 18 states and Union Territories of India. There were 10 confirmed hMPXV cases, among them five cases (males from Kerala) had a travel history from the United Arab Emirates to India. All the cases were immunocompetent with no comorbidities, had a mean age of 31 years, and presented with a short prodromal phase of fever, myalgia, and vesiculopustular rash primarily in the genital area, face, trunk, and extremities [26].

Clinical presentation of hMPXV

hMPXV infection was thought to resemble smallpox in terms of symptoms, severity, and mortality, however, it was associated with low transmissibility between human beings and less mortality compared to smallpox. After cessation of smallpox vaccination, younger people (40 to 50 years, depending on the country) may be more susceptible to mpox [28,21].

Mpox is usually a self-limited disease, with symptoms lasting from two to four weeks. The incubation period of mpox usually ranges from five to 21 days [29]. Observational studies in the mid-1980s showed an incubation period of 10-14 days while another study done in cases with clear exposure suggested a median incubation period of seven days with a range of three to 20 days [30,21]. In recent outbreaks, the case fatality rate has been around 3-6% [29]. Milder cases may be undetected and represent a risk of person-to-person transmission [22].

A characteristic two-day prodrome, followed by fever and malaise, occurs in most patients before the development of the rash. About 90% of patients infected with mpox develop lymphadenopathy which can be unilateral or bilateral and occurs in the submandibular, cervical, postauricular, axillary, or inguinal lymph nodes, or any combination of these. As lymphadenopathy is not a characteristic feature of smallpox, this clinical finding became a key distinguishing feature of hMPXV [31,32].

**Figure 3:**
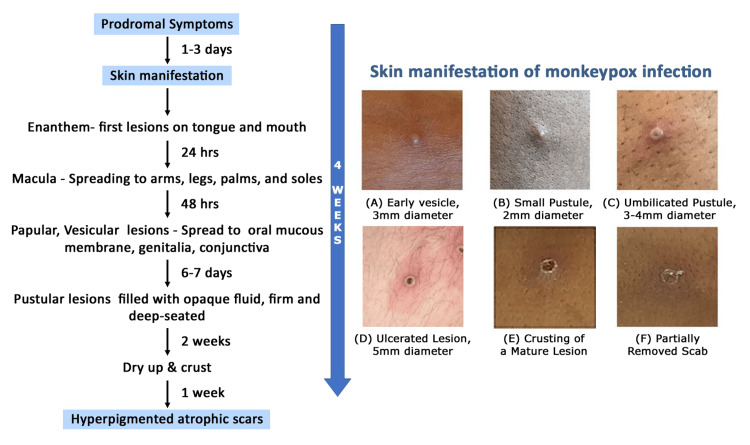
Evolution of skin rash in monkeypox infection The figure illustrates the development of the skin lesions in monkeypox from a maculopapular rash to vesicles, pustules, and eventual scab formation over a period of 10-14 days [30,33]. Source: Centers for Disease Control and Prevention (CDC). (Permission has been obtained.)

The typical hMPXV rashes begin as maculopapular lesions of 2-5 mm in diameter. Reports from African outbreaks suggest that the rash becomes generalized in distribution in most cases, spreading in a centrifugal pattern. The total lesion burden at the peak of the rash can be quite high (>500 lesions) to relatively less (<25). The skin lesions typically progress to papule, vesicle, pustule, and crust over a period of 14-21 days, before sloughing and leaving pigmented scars [28,33] (Figure 3). 

The skin manifestation depends on age, vaccination status, nutritional status, and associated other infections like Human Immunodeficiency Virus (HIV). In addition to smallpox and chickenpox, the differential diagnosis of a vesiculo-papular rash includes drug eruptions, eczema herpetic, dermatitis herpetiformis, rickettsialpox, and molluscum contagious [33,21].

Young children and immunocompromised persons, including persons with HIV infection, have been reported to be at increased risk for severe outcomes [21,28,29]. In cases of advanced immunosuppression, a severe necrotizing form of monkeypox seems to mirror the clinical characteristics of an acquired immunodeficiency syndrome (AIDS) defining condition. This includes a notable prevalence of aggressive dermatological and systemic manifestations, often leading to fatal outcomes [34].

Complications of mpox include pneumonitis, encephalitis, keratitis, and secondary bacterial infections [21,28]. Analyzing 528 cases of hMPXV across 16 countries from April to June 2022, it was observed that 13% of patients were hospitalized with complications such as anorectal pain, myocarditis, superimposed soft tissue infection, or acute kidney injury. Children and as well pregnant females are at higher risk of developing complications [21]. Mpox during pregnancy may lead to congenital mpox or stillbirth [35]. Clinical cases of hMPOX can be confirmed by the detection of viral DNA by polymerase chain reaction (PCR) from the samples taken directly from rash, fluid, or crusts during the symptomatic phase [36].

Treatment approach for hMPOX

The hMPXV is self-limiting like other viral infections. The primary care and symptomatic management are enough for mild to moderate hMPXV cases [37]. According to Centers for Disease Control And Prevention (CDC), the treatment of hMPXV is considered only for severe disease presentation or those who are at high risk of developing the severe disease (immunocompromised conditions, e.g., HIV/AIDS, leukemia, lymphoma, generalized malignancy, etc.) or mpox virus aberrant infections in vulnerable group (pediatrics, pregnant and breastfeeding mother) or accidental implantation in eyes, mouth, the genitals or anus) [38]. Supportive care and hospitalization may be required for those who need more intensive pain management or who have or are at risk for dehydration (e.g., nausea, vomiting, dysphagia, severe tonsillitis) [12,37]. The Ministry of Health and Family Welfare (MoHFW), Government of India released a comprehensive guideline for the management of monkeypox disease in July 2022 [39,37].

Antiviral Drugs

Currently, there is no specific licensed or Food and Drug Administration (FDA) approved drugs available for treatment of hMPXV infection. There are no observational studies or published trials for the treatment of hMPXV infection [40]. The efficacy of tecovirimat to treat mpox has not been fully evaluated in humans as limited scope for conducting clinical trials in the region where mpox is endemic [41]. The efficacy of tecovirimat to treat mpox has not been fully evaluated in humans as limited scope for conducting clinical trials in the region where mpox is endemic [42]. The treatment depends on repurposed FDA-approved drugs [43]. Because of the genetic similarity between mpox and smallpox viruses, the antiviral drugs and vaccines developed for smallpox may be used to treat and prevent hMPXV infection [44]. The therapeutic medical countermeasures (MCMs) are FDA-regulated drugs and biologics that have shown effectiveness against other orthopoxviruses and have been used to treat severe hMPXV. Tecovirimat (under the commercial name TPOXX or ST-246), brincidofovir (BCV), and vaccinia immunoglobulin intravenous (VIGIV) are the MCMs available as therapeutic options for the treatment of hMPXV [44].

Two antiviral drugs, tecovirimat, and brincidofovir were approved under a regulation of “animal rule” (efficacy is established based on adequate and well-controlled studies in animal models of the human disease) [45,46].

Tecovirimat is an inhibitor of viral p37, an envelope-wrapping protein. It inhibits the formation of viral envelopes and their subsequent release from infected cells [47]. In preclinical testing in the animal models (nonhuman primates) for smallpox, the group with tecovirimat exhibited a significantly higher survival rate compared to the placebo. In clinical studies, tecovirimat shortens the duration of illness, hastens the virus shedding, and is well tolerated by patients. Tecovirimat has been used in travelers returning from Nigeria and in patients who acquired mpox during the outbreak that began in May 2022 [48,37]. Use of tecovirimat for the treatment of mpox in the United States is permitted only through an FDA-regulated Expanded Access Investigational New Drug (EA-IND) mechanism. CDC holds a nonresearch EA-IND protocol that facilitates access to and use of tecovirimat for the treatment of mpox [41].

Two dosage formulations, capsules (200 mg) for oral and solution for intravenous administration (single dose vial of 200 mg in 20 mL) were approved by the FDA in July 2018 and May 2022 respectively [44,45]. The recommended dose in adults and children weighing 40 to 120 kg is 600 mg twice daily for 14 days. For adults weighing more than 120 kg, three times daily administration, and for small children, a dose based on body weight is recommended. Longer-term therapy is not recommended. Tecovirimat is generally well-tolerated with mild adverse events such as headaches, myalgia, fatigue, gastrointestinal upset, nausea, and diarrhea. Rare but potentially severe adverse events include hypersensitivity reactions, rash, and embryofetal injury [47]. Out of a total of 549 hMPXV cases treated with tecovirimat under an EA-IND protocol, only 3.5% adverse events were reported which were mild in nature [37]. Parenteral administration of tecovirimat should be cautiously in patients with renal impairment (contraindicated if the creatinine clearance is < 30 mL/min) and pediatric patients < 2 years of age (as they have an immature renal tubular function) [36,49]. Animal data shows no fetotoxicity but no human data are available regarding the impact on the fetus or its presence in breastmilk [45]. Tecovirimat is a weak inducer of cytochrome P450 (CYP)3A and a weak inhibitor of CYP2C8 and CYP2C19. The repaglinide (increased levels causing hypoglycemia) and decreased midazolam levels. Tecovirimat increases the level of repaglinide, decreases the level of midazolam, and may also interact with antiretroviral drugs, doravirine, rilpivirine, and maraviroc, but the effects are mild, and dose adjustments may not be required. Tecovirimat should not be used concomitantly with carbotegravir/rilpivirine. Tecovirimat has a low barrier to resistance and change in the VP37 protein may decrease the efficacy of the drug [50]. Tecovirimat can be considered for prophylactic use in an exposed person with severe immunodeficiency in T-cell function for which smallpox or mpox vaccination following exposure to mpox virus is contraindicated [51].

Clinical Trials of Tecovirimate

A limited number of clinical trials have been registered on ClinicalTrials.gov focusing on antiviral drugs (tecovirimat) and vaccines for testing efficacy and safety in hMPXV infection since the first case of mpox was reported [52]. Currently, a randomised trial is underway to study the effectiveness of tecovirimat in the hMPOX outbreak [53]. Another phase 3 trial “Study of Tecovirimat for Human Monkeypox Virus (STOMP) is ongoing for testing the efficacy of oral tecovirimat for the treatment laboratory-confirmed or presumptive hMPXV cases [54]. The National Institute of Allergy and Infectious Diseases (NIAID), part of the U.S. National Institutes of Health and the DRC’s National Institute for Biomedical Research (INRB) are co-leading a clinical trial for evaluating the effectiveness of tecovirimat in adults and children with mpox in the Democratic Republic of the Congo (DRC) under PALM (“Pamoja Tulinde Maisha” a Kiswahili phrase that means “together save lives”), a government-to-government partnership [55]. NIAID funded the preclinical studies for tecovirimat to explore the mechanism of action, safety, and efficacy and subsequently, NIAID together with the Biomedical Advanced Research and Development Authority (BARDA), part of the U.S. Department of Health and Human Services, funded Phase 1, and Phase 2 clinical trials to test the safety and pharmacokinetics of an oral formulation of the tecovirimat [55].

Brincidofovir was approved by the FDA in June 2021 for the treatment of smallpox infection in adult and pediatric patients including neonates [56]. Brincidofovir (BCV) is a lipid conjugate of cidofovir. Cidofovir has in vitro activity against mpox and is effective against the lethal monkeypox challenge in animal models, but it is associated with significant adverse events including nephrotoxicity [57]. BCV is an oral formulation of cidofovir with an improved safety profile and less renal toxicity [58]. BCV is an OPXV nucleotide analogue, DNA polymerase inhibitor. The recommended dose of BCV is 200 mg (2 tablets of 100mg) once weekly for 2 doses (on Days 1 and 8). BCV can cause gastrointestinal side effects and requires monitoring of liver enzymes. BCV is potentially teratogenic and should not be used in pregnancy [58]. Clinical data regarding BCV in hMPXV infection is lacking currently [57].

Topical ophthalmic preparation of trifluridine is recommended for ocular involvement in cases of inadvertent viral inoculation in the eye [59]. Trifluridine is a nucleoside analog structurally similar to thymidine, being incorporated into viral DNA, interfering with DNA synthesis and further replication. Trifluridine has activity against HSV-1, HSV-2, and vaccinia viruses [60].

Limitation of drug trials for mpox infection: Animal studies using variola virus, including nonhuman primate models, are not consistently reproducible and require unnaturally high viral challenge doses; moreover, variola virus infection in animals does not mimic human smallpox disease [44].

Immunoglobulin therapy

Vaccinia immunoglobulin intravenous (VIGIV, CNJ-016) was first introduced for clinical use to treat the complications of smallpox vaccinations in 2005 after extensive clinical studies demonstrated by Kempe et al. [61]. Experimental results in animal models support the role of VIGIV in prophylaxis and treatment of OPXV infections in animals with a functioning immune system [62,63]. CDC holds an expanded access protocol that allows the use of VIGIV for the treatment of OPXV (including monkeypox) in 2022 during the outbreak [64]. The use of VIGIV is restricted only to adults and children with immunocompromised status or those who are ineligible for antiviral treatment (including tecovirimat) or after antiviral treatment has been exhausted or in conjunction with antiviral and/or other therapies for severe manifestations. The dose of VIGIV based on actual body weight, 6,000 U/kg is administered intravenously as soon as symptoms appear. The use of VIGIV is justified based on the clinical judgment and individual risk-benefit assessment. Use of VIGIV in neonates may be considered as PEP in the neonates as there is a lack of safety and effectiveness data regarding vaccine (JYNNEOS) and antiviral drugs(tecovirimat) use in neonates [65]. 

Immunomodulators

Monoclonal antibodies have been widely suggested for the treatment of orthopoxvirus infections, which can be a potential therapeutic strategy for severe mpox infection [65,62]. The in vitro experiment showed that Interferon βeta (IFN-β) markedly inhibits the production and spread of mpox. Recombinant IFN-β has the potential to be a novel and safe agent for the treatment of monkeypox disease [66]. Future approaches may include the development of specific monoclonal antibodies (MAb) to increase VIGI’s potency and/or specific anti-poxviral drugs might improve outcomes of patients with severe combined immunodeficiencies [62].

Preventive Measures

Primary prevention involves implementing measures to prevent the initial occurrence and transmission of the disease such as isolation, care of personal hygiene, avoiding direct contact with suspected cases or animals, and using personal protective equipment [36,37].

Vaccination

After the eradication of the smallpox and cessation of vaccination, the mpox has emerged as the most important poxvirus of public health concern [17]. The recent spread of monkeypox cases in non-endemic countries is likely due to the absence of OPXV immunity among these populations [67]. As per active surveillance data of DRC, the risk of hMPXV is inversely associated with smallpox vaccination, and reported that individuals who were vaccinated had a 5.21-fold lower risk of mpox infection as compared with unvaccinated persons [68]. However, seven cases of mpox infection reported in the UK had no history of pre-exposure to smallpox vaccination [40]. In a review of several observational studies, the WHO reported that vaccination against smallpox is 85% effective in preventing monkeypox [69]. Comparison of mpox virus genome sequences in the 2022 outbreak to those of vaccinia virus suggests that vaccinia virus-based vaccines induced immunogenicity which was highly cross-reactive against mpox virus [68]. As per WHO recommendation, targeted vaccination is recommended only for those who have a high risk of exposure including health workers, lab workers, and those with multiple sexual partners. Mass Vaccination against monkeypox is not recommended [70].

Two vaccines (JYNNEOS and ACAM2000) were approved by the FDA on August 9, 2022, for immunization against smallpox disease and mpox in the United States and Australia. It has been made available under an exemption provided by section 18A of the Therapeutic Goods Act 1989 [71].

No data are available on the clinical efficacy or effectiveness of JYNNEOS or ACAM2000 vaccines in the current outbreak. The limited data on the effectiveness of the JYNNEOS vaccine in the current outbreak are becoming available after interim clinical considerations for the use of JYNNEOS and ACAM2000 vaccines during the 2022 United States mpox outbreak [72]. 

JYNNEOS is a live attenuated virus vaccine produced from the Modified Vaccinia Ankara-Bavarian Nordic (MVA-BN) non-replicating OPXV strain. JYNNEOS is approved in the United States. It was licensed by the FDA in September 2019, a single dose of 0.5 ml subcutaneously for the prevention of smallpox and monkeypox disease in adults ≥18 years of age and older who are at high risk for smallpox or monkeypox infection. Minor adverse effects (local reaction and headache, tiredness, nausea, chills, and muscle aches) were observed following vaccination [73]. Historical data have shown that smallpox vaccination with vaccinia virus was approximately 85% effective against monkeypox [4]. The vaccine is approved in Europe for smallpox as IMVANEX, although the UK has been using it off-label in response to mpox cases [74]. Caution is required in people who have a history of a severe allergic reaction to a component of the vaccine (e.g., gentamicin, ciprofloxacin, chicken or egg protein) or an acute illness. There is no risk of inadvertent inoculation and autoinoculation [75]. There is insufficient data to determine the risks of these vaccines in pregnant and breastfeeding women. However, the vaccine can be offered to pregnant and breastfeeding women who are otherwise eligible, after a discussion of the risks and benefits and using shared decision-making [76]. Safety data in children are limited. However, one study found that a single dose of MVA-BN was well-tolerated [77].

ACAM2000 is a live, replicating vaccinia virus vaccine, licensed by the FDA in August 2007 for active immunization against smallpox disease and now it has been approved during the Mpox outbreak under an EA-IND protocol. ACAM2000 is given as a series of two doses, 28 days apart [78]. Few serious side effects were observed following ACAM2000 vaccination like myocarditis and/or pericarditis (among one in every 175 persons, who got the vaccine for the first time), swelling of the brain or spinal cord, infection at the vaccination site or accidental infection of the eye with the vaccine virus [73]. Contraindications include history of severe allergic reaction (e.g., anaphylaxis) after a previous dose of the vaccine, atopic dermatitis, other exfoliative skin conditions, immunosuppression (congenital or acquired), history of drugs or treatments that cause immunosuppression, HIV infection (regardless of immune status), transplant recipients, autoimmune disease, eye disease being treated with topical corticosteroids, pregnancy and breastfeeding, age <1 year, allergy to vaccine component and underlying heart disease or cardiac risk factors. Vaccine adverse events may be managed with vaccinia immune globulin and antiviral agents [79]. 

Another third-generation vaccine is LC16 m8, an attenuated vaccinia virus derived from the Lister (Elstree) strain that has been licensed for active smallpox immunization in Japan since 1975. This vaccine was currently expanded by Japan to include protection against monkeypox. These third-generation vaccines have a higher safety profile due to their attenuated phenotype and can be given to immunocompromised people [80]. The safety and effectiveness of LC16m8 against MPXV were evaluated in a study involving 50 healthy adults in Japan. The results showed that it elicited neutralizing antibody responses without any significant safety issues [81].

Pre-exposure Prophylaxis

The WHO recommends pre-exposure vaccination with appropriate approved vaccines (e.g., JYNNEOS/MVA-BN, ACAM2000, LC16) for people who are at high risk of exposure including clinical laboratory and healthcare personnel performing diagnostic testing for mpox and outbreak response team members as designated by national public health authorities [82].

As per a meeting held on October 2023, the Advisory Committee and Immunization Practices (ACIP) recommends vaccination for persons ages ≥18 years known to be at risk for mpox, including gay, bisexual, and other men who have sex with men, transgender, or nonbinary people who in the past six months have had a new diagnosis of ≥1 sexually transmitted disease or more than one sex partner or sex at a commercial sex venue or sex in association with a large public event in a geographic area where mpox transmission is occurring [83].

Post-exposure Prophylaxis

The WHO recommends postexposure vaccination with an appropriate vaccine for contacts of cases, ideally within four days of first exposure (and up to 14 days in the absence of symptoms). Postexposure vaccination is recommended for contacts with higher-risk exposures and may be considered for contacts with intermediate-risk exposures with special risk populations. Post-exposure prophylaxis (PEP) is considered on an individual case-by-case basis. The CDC has created informed guidance to assess the risk of exposure and make informed decisions with regard to the monkeypox vaccine PEP [84]. PEP is the most effective for preventing monkeypox infection for individuals with risk factors if the vaccine is administered within four days of exposure; after four to 14 days, vaccination may help reduce symptoms only, but not prevent the infection from developing [72]. As the disease was eradicated in 1978, the smallpox vaccination ceased in India. Requirements of vaccination or “emergency procurement of monkeypox vaccine” have not been initiated currently, considering the situation is very much under control in India [69]. Since vaccination with the vaccinia virus vaccine is contraindicated in patients with severe immunodeficiency in T-cell function, such patients with an exposure history may be given VIGIV as post-exposure prophylaxis [85]. Clinical studies conducted by Kempe et al. concluded that post-exposure smallpox vaccination along with VIGI conferred better protection than vaccination alone [63].

Monkeypox vaccination in India: Smallpox vaccination ceased in India as the disease was eradicated in 1978. Requirements of vaccination or “emergency procurement of monkeypox vaccine” have not been initiated currently considering the situation is very much under control in India [37]. 

Way forward: new drug targets for mpox

There are molecules that are effective against the vaccinia virus. Resveratrol, a natural polyphenol found in grapes, berries, etc., has also been shown in vitro to significantly reduce mpox replication (both clades) by suppressing DNA synthesis and associated downstream gene expression. The results will prompt further investigation of its effect on other poxvirus replication steps as well as the mechanism to inhibit vaccinia virus (VACV) replication. The identification of resveratrol as a VACV DNA synthesis inhibitor may allow for developing alternative or compensative strategies to better manage current and re-emergent poxvirus infections and complications caused by poxviruses-based therapeutics [86].

Using a high throughput virtual screening approach, Srivastava et al. identified 15 triple-targeting FDA-approved drugs that can inhibit three viral targets, including topoisomerase1, p37, and thymidylate kinase [43]. Further, the molecular dynamics simulation analysis of the top hits such as Naldemedine and Saquinavir with their respective targets reveals the formation of stable conformational changes of the ligand-protein complexes inside the dynamic biological environment. So these triple-targeting molecules can be used to develop an effective therapy for the currently spreading mpox [43].

## Conclusions

FDA-repurposed antiviral drugs and vaccines are used to treat severe human monkeypox virus (hMPXV) infection. Currently, this treatment approach is justified by the research done on animal models. The role of mpox-specific therapeutics remains experimental and can be used under expanded access protocols. Recently clinical trials are underway to establish therapeutic effectiveness for hMPXV cases. Monoclonal antibodies, IFN-β, resveratrol, and 15 triple-targeting FDA-approved drugs are the potential new drug targets that need to be explored. The pre- and post-exposure prophylaxis with vaccinations, are being implemented within high-risk groups to mitigate the spread of this disease.
